# Evaluation of a Case Management to Support Families With Children Diagnosed With Spinal Muscular Atrophy—Protocol of a Controlled Mixed-Methods Study

**DOI:** 10.3389/fped.2021.614512

**Published:** 2021-08-03

**Authors:** Jana Willems, Erik Farin-Glattacker, Thorsten Langer

**Affiliations:** ^1^Section of Healthcare Research and Rehabilitation Research, Faculty of Medicine and Medical Center - University of Freiburg, Freiburg, Germany; ^2^Department of Neuropediatrics and Muscle Disorders, Center for Pediatrics, Faculty of Medicine, University of Freiburg, Freiburg, Germany

**Keywords:** case management, family-reported outcomes, care integration, participatory research, rare neuromuscular disorders, spinal muscular atrophy

## Abstract

**Background:** Spinal muscular atrophy (SMA) is a rare neuromuscular disease characterized by degeneration of the anterior horn cells in the spinal cord, resulting in muscle atrophy, and proximal muscle weakness. SMA presents with a wide range of symptoms requiring multiple clinical specialists and therapists. Integrating care between disciplines can be challenging due to the dynamic course of the disease, and great distances between specialist centers and local providers. Insufficient care integration can lead to suboptimal quality of care and more difficulties for patients and families. This study aims to improve care integration through a Case Management intervention, and taking a mixed-methods approach, to evaluate its impact.

**Methods:** An exploratory, controlled, two-armed study with baseline, post- and follow-up measurement and process evaluation is conducted to evaluate our intervention compared to usual care. Through a multi-perspective state analysis, we investigate the experiences of caregivers and healthcare providers concerning the actual healthcare quality of patients with SMA I and II. Semi-structured interviews and care diaries are used. We apply that data to conceive a tailored Case Management intervention supplemented by a digital platform. The intervention's effect is examined in comparison to a control group taking a mixed-methods approach. As primary endpoints, we investigate the caregivers' health-related quality of life and the quality of care integration. Secondary endpoints are the use of healthcare services (patients and caregivers) and costs. We assess the process quality from the perspectives of caregivers and healthcare providers through semi-structured interviews.

**Discussion:** This is an exploratory, controlled study to assess the impact of a tailored Case Management intervention to improve the care of patients with SMA I and II. After the evaluation, results on feasibility, expected effect sizes, and process quality will be available. On this basis, future randomized controlled trials can be planned. If demonstrated beneficial, the experience gained within this study may also be valuable for care strategies in other regions and other (non-pediatric) patient groups with rare diseases and/or chronic, complex conditions.

**Clinical Trial registration:**https://www.drks.de/drks_web/navigate.do?navigationId=trial.HTML&TRIAL_ID=DRKS00018778, identifier: DRKS00018778.

## Background

### Spinal Muscular Atrophy

SMA is a genetic, neuromuscular disease characterized by degeneration of alpha motor neurons in the spinal cord due to loss or dysfunction of the SMN-1 gene 5q11-q13 (1). The incidence is 1:6.000 to 1:10.000 births/year, making it the most frequent genetic cause of death in childhood (2). SMA covers a disease spectrum with varying degrees of severity. A classification into subtypes is defined according to patient age at initial manifestation, and best-achieved motor function (1). Phenotypes are divided into SMA I-IV, whereas SMA I is the most severe and most frequent subtype (1). Disease onset in infancy is associated with a significant reduction in life expectancy (3). Patients with SMA I are the most severely affected; they never learn to sit unaided. In SMA II patients, motor development stagnates after achieving head control and free sitting, while patients never learn to walk unaided (1). The present study protocol refers to the care of patients with SMA I and SMA II.

### Healthcare Situation of Patients With SMA I and II

Despite significant improvements in the pharmacological treatment, e.g., by Nusinersen (4) or Onasemnogene abeparvovec-xioi (5), SMA has remained a chronic complex condition for the majority of patients over the past years (2). Due to these treatment options, the life span and course of the disease in these patients will change in yet unknown ways, requiring a revision of existing care models (6). The focus is mainly on improving health-related quality of life through a multidisciplinary, symptomatic care concept, which is frequently accompanied by the need for home respiration, tube feeding, and acute care as well as orthopedic and rehabilitative interventions (7–10). Treatment recommendations are outlined in a consensus-based standard-of-care concept (1, 11). The recent availability of new therapies offers a promising perspective for further changes in treatment and the associated prognosis (11).

### Impact on the Lives of Affected Families

Far-reaching effects on caregivers' lives arise when a child is chronically ill. Caregivers often assume extensive responsibilities in their child's daily care and care coordination. The demands of care and dealing with this progressive disease influence the emotional and social life of the whole family and lead to various psychosocial and economic challenges (7–13). The severity of challenges varies with the phenotype of SMA; caregivers of children with SMA I have reported the most severe psychosocial effects (14). In addition, caregivers of children with SMA often face challenging decisions possibly affecting their child's quality and length of life. These decisions include multiple therapeutic options, such as tube-feeding, continuous ventilation (15, 16), medical treatment, etc. In addition to the high dynamic care needs of the children, there is also a lack of clarity regarding the right timing of interventions to achieve a maximum response or the desired therapeutic effect. Preclinical and clinical data associate the earliest possible use of interventions with better outcomes (17). This resulting pressure to act increases the challenges on families while the long-term consequences remain uncertain (18).

### Quality of Care From the Perspective of Caregivers of Patients With SMA I and II

Some studies describe how caring for a child with SMA affects families. However, relatively few studies provide information on how affected caregivers perceive the quality of their child's care (19). In some studies, caregivers report support from healthcare professionals, involvement in their child's treatment (7, 20), and related participatory decision-making (6). At the same time, there are studies in which caregivers indicate dissatisfaction with care coordination (21) and care integration (8). *Care coordination* refers to the quality of interaction between patients/caregivers, healthcare professionals, providers of medical aids, health insurance companies, and other services (22). Good care coordination is a precondition for good care integration. To be able to react flexibly to the needs of patients and families, the care system must be able to adapt to the respective situation. The term *care integration* therefore also contains the element of patient-centeredness (8) and describes the quality of coordinated activities from the perspective of patients and families (19). Healthcare is often fragmented between sectors and disciplines in the German healthcare system (23). This affects patients with chronic and complex conditions in particular, as they depend on services from different healthcare disciplines (8, 24). Patients with rare diseases often require specialized and multidisciplinary treatment, usually provided by specialized centers. At the same time, they also have needs that can be met by non-specialized care providers close to their homes. Great distances between home and specialized neuromuscular centers often interfere with prompt and effective communication between families and healthcare providers. Because of many different treatment settings, there is often confusion about who bears “overall responsibility” for the care of a chronically ill child (19). Interdisciplinary and intersectoral cooperation between the various medical and non-medical care providers is therefore of high importance (25, 26), but often not available (27). The coordination of complex therapy measures constitutes a major factor in the success of treatment interventions. Affected caregivers often find themselves in the role of coordinator and emphasize their initiative within care management (19, 28), which can be additionally distressing (24, 29). It is indisputable that caregivers are considered experts in their child's health, but the healthcare system's support is needed to enable them to cope with their child's care (30).

### Case Management

To meet these challenges, continuity of care seems particularly important. This can be accomplished by a specialized care team or a permanent contact person during the entire course of the disease (7). In this project, we propose a Case Management intervention (in the following: CM) as “[…] case-oriented interdisciplinary coordination of care regardless of the professional, hierarchical or institutional assignment” is suitable for supporting families within care management (31). The approach of a patient/family-related organization in the care context enables long-term assistance and needs-oriented networking on an individual level (32). The tasks of CM include a comprehensive provision of information, integration between the patient's multidisciplinary healthcare providers, and psychosocial support for the family, with the emphasis on high-quality, cost-effective care (31, 33, 34). Overall, it should contribute to enhancing the patient-orientation of care and strengthen the caregivers in their self-management. Pediatricians also advocate more frequent use of CM (35). A coordinator as a link between caregivers and professionals can have a positive impact on both parties: Caregivers feel comfortable with their child's care, and professionals benefit from an effective communication partner (24).

The use of a CM program can result in raising the perceived quality of care (34, 36) and lowering the demands on caregivers (34). There is evidence from the UK that a “coordination service” for rare disease patients leads to positive results from the perspective of those affected, even though no controlled evaluation study was conducted and the coordination support was not disease-specific (37). CM in adult cancer care as individual support in the care system for patients and their families has proved to have an impact on the quality of care and well-being of those involved (38).

IT support within CM can ensure the timely availability of information. This is a central aspect to optimize care processes and exploit the available resources in a more targeted way (39). Patient-centered e-health services also offer the possibility of a cross-sectoral communication interface (40).

The current care situation of children with SMA I and II reveals the need for holistic and sustainable healthcare that incorporates central, active participation of the patients and their caregivers in care management, as well as a continuous dialogue between healthcare professionals and families (8, 39). To adapt the care of children with SMA I and II to the needs of families implies that we must pay closer attention to the experiences of caregivers, and their coordination between different care settings (19). However, to design integrated care models for children with SMA I and II in a needs-oriented framework, it is also necessary to include the perspectives of the multi-professional care team involved (10). In this paper, we describe the development of a concept and an evaluation study of a CM approach tailored to the needs of patients with SMA I and II and their caregivers.

## Methods/Design

**Figure 1:**
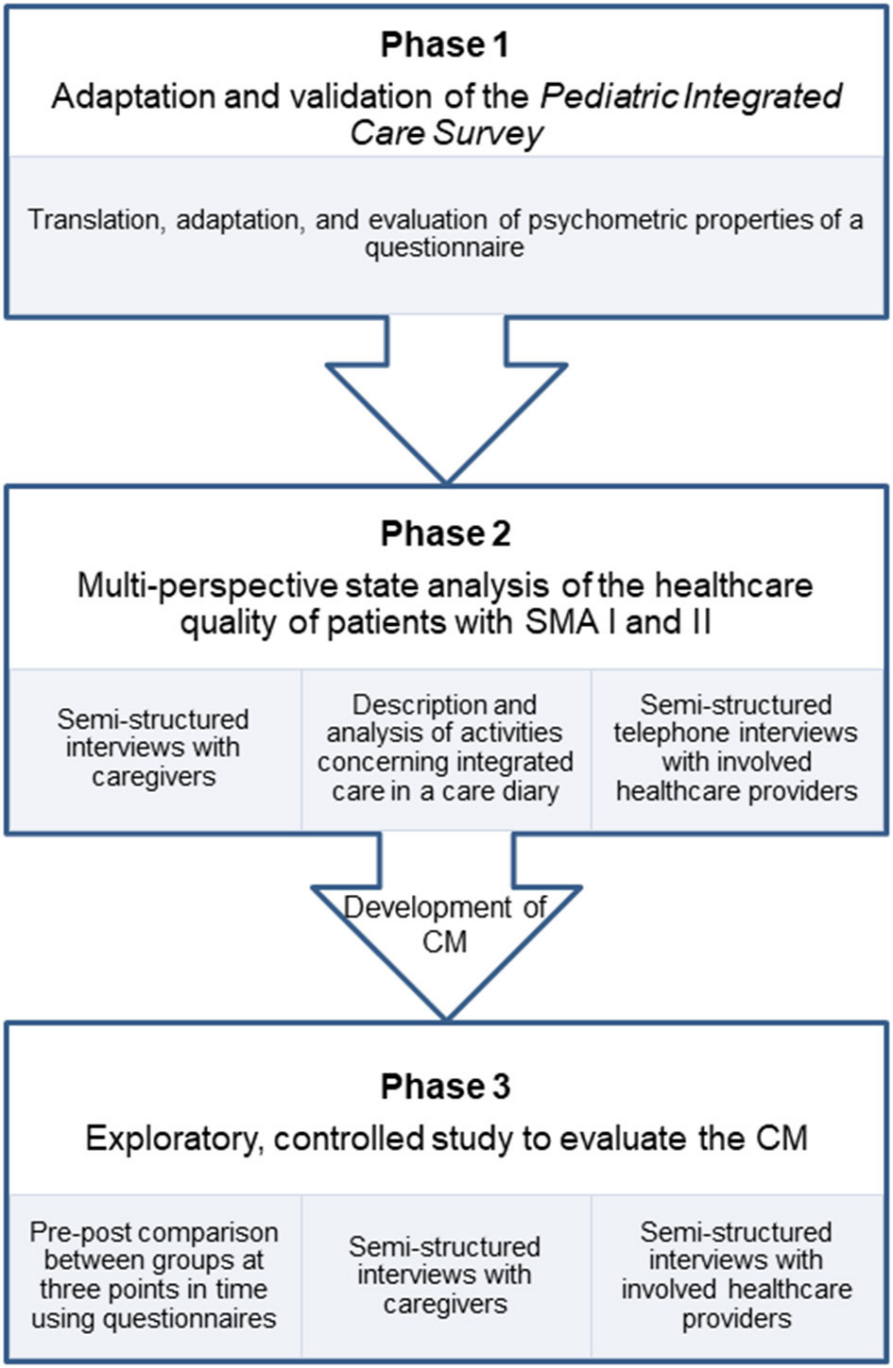
Flow chart of the three phases of the study.

In this paper, we present the study protocol of the “SMA-Case Management-Project” (SMA-C+). The study is divided into three successive phases, which consecutively depend on each other (Figure 1). We apply a mixed-methods design in which we use quantitative and qualitative methods for data collection and analysis. The aim of this study is three-fold:

- Psychometric testing of a patient-reported outcome measure to assess the quality of care integration in pediatrics. To date, no such instrument is available in German. We therefore adapt and validate the *Pediatric Integrated Care Survey* (41). The questionnaire's psychometric properties are evaluated by testing construct validity, content validity, and internal consistency. The instrument will be used to assess measure the effect of the CM from the caregivers' perspectives. We will refer to this part of the study as *PICS* testing and phase 1.
- Analyzing the experiences of care in patients with SMA I and II from the perspectives of caregivers and care providers. A good understanding of the different stakeholders' perspectives is necessary to develop a CM, which is responsive to the needs of caregivers and care providers, and is capable to improve the quality of care integration. We will refer to this part of the study as multi-perspective state analysis and phase 2.
- Assessing the impact of CM on the quality of care integration. We conduct an exploratory, prospective, controlled, two-armed study involving baseline, post- and follow-up measurements, and process evaluation. We will refer to this part of the study as main study and phase 3.

### Study Design, Setting, and Recruitment

The intervention study of the SMA-C+ project is designed as an exploratory, prospective, controlled, two-armed study involving baseline, post- and follow-up measurements, and process evaluation. This study is being conducted by the Medical Center—University of Freiburg, Germany (Department of Neuropediatrics and Muscle Disorders and Section of Healthcare Research and Rehabilitation Research, SEVERA). The following centers and institutions support the recruitment of study participants: the Department of Neuropediatrics and Neuromuscular Center for Children and Adolescents, University of Duisburg-Essen, Essen; Department of Child Neurology, Children's Hospital, University of Tuebingen, Tuebingen; the Federal Working Group of Social Pediatric Centers (BAG-SPZ) of the German Society for Social Pediatrics and Youth Medicine; the patient organization “Initiative SMA,” Munich; and the “Children's Network,” Berlin. The study began in February 2019 and will continue until the second half of 2022.

#### Phase 1—PICS Testing

The questionnaire on integrated pediatric care was developed by the Boston Children's Hospital to measure the experience of families reporting on the care integration of child health. The original English version of the PICS has well-established psychometric properties (41). It should be modified for the assessment of integrated care in Germany, as many items are closely linked to the American healthcare system. The questionnaire's psychometric properties are evaluated by testing construct validity, content validity, and internal consistency. To ensure validation across a large spectrum of conditions, caregivers of children with a chronic disease (not exclusively SMA) are included. A case number of *N* = 400 caregivers is being targeted. Caregivers of children with a chronic disease are being recruited nationwide *via* personal contact from several social pediatric centers (SPC). SPCs are interdisciplinary outpatient clinics that offer multidisciplinary care for children with complex healthcare needs and special psychosocial difficulties. The participating SPCs are located in Bochum, Bremen, Celle, Freiburg, Lübeck, Rotenburg (Wuemme), and Stuttgart. In addition, the “Children's Network Germany” patient organization posted an invitation regarding this study on its website. Participants receive a 20 € voucher per completed questionnaire.

#### Phase 2—Multi-Perspective State Analysis

A sample of *N* = 40 caregivers and *N* = 40 healthcare providers each is targeted. Caregivers are included if they have a child with a genetically confirmed SMA type I or type II. Participation is independent of the length of the child's disease history, i.e., inclusion is possible immediately after diagnosis or at a later stage.

Caregivers are being recruited if their children receive treatment at one of the following centers: the Center for Neuromuscular Diseases of Freiburg, the Essen Center for Rare Diseases and the Department of Child Neurology of Tuebingen. Potential participants are being continuously identified and recruited through personal contact with the treating neuropediatricians in the neuromuscular centers. Eligibility criteria are controlled during the recruitment process.

To identify the corresponding healthcare providers, participating caregivers are asked to provide contact information on the person most involved in caring for their children, and an agreement is signed releasing that person from their medical confidentiality obligation. Selected healthcare providers are being recruited *via* personal contact through neuropediatricians in the neuromuscular centers with information on the study. Healthcare providers receive a 75 € voucher for participating in an interview.

#### Phase 3—Main Study

Caregivers of children with SMA I and II (25 participants in intervention and control group, respectively) are being enrolled. Participants in the intervention group are being recruited through the neuromuscular center Freiburg. Control-group participants are being recruited at the Essen Center for Rare Diseases and the Department of Child Neurology of Tuebingen. In addition, the patient organization “Initiative SMA” patient organization supports recruitment through public relations (e.g., online posting). The control group receives current practice (“usual care”), complying with the SMA standard of care (12).

Participants in the intervention group receive a fixed sum of 60 € at the end of the study, whereas participants in the control group receive 25 € per interview or questionnaire completed.

**Figure 2:**
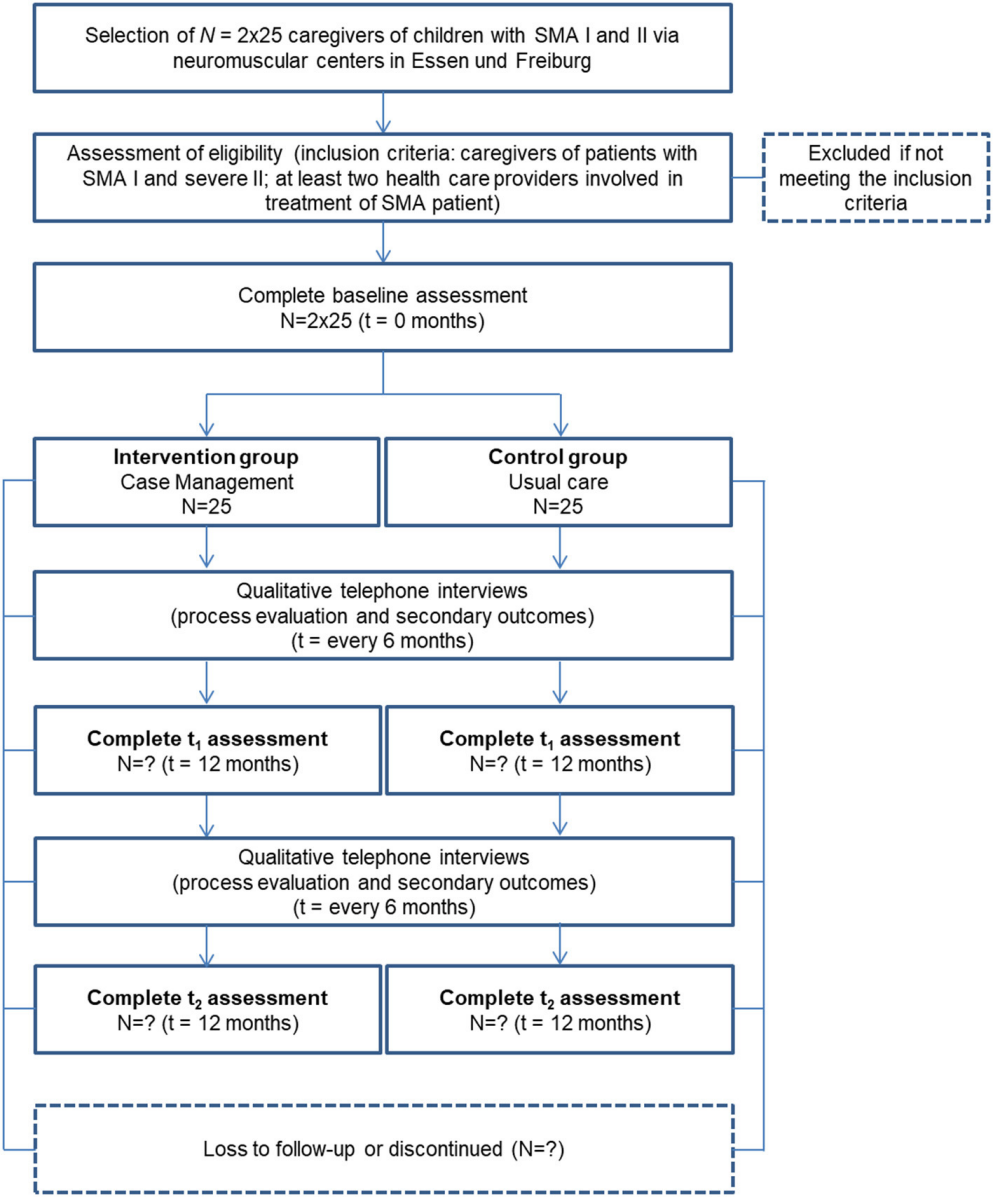
Participant timeline of caregivers in phase 3.

**Figure 3:**
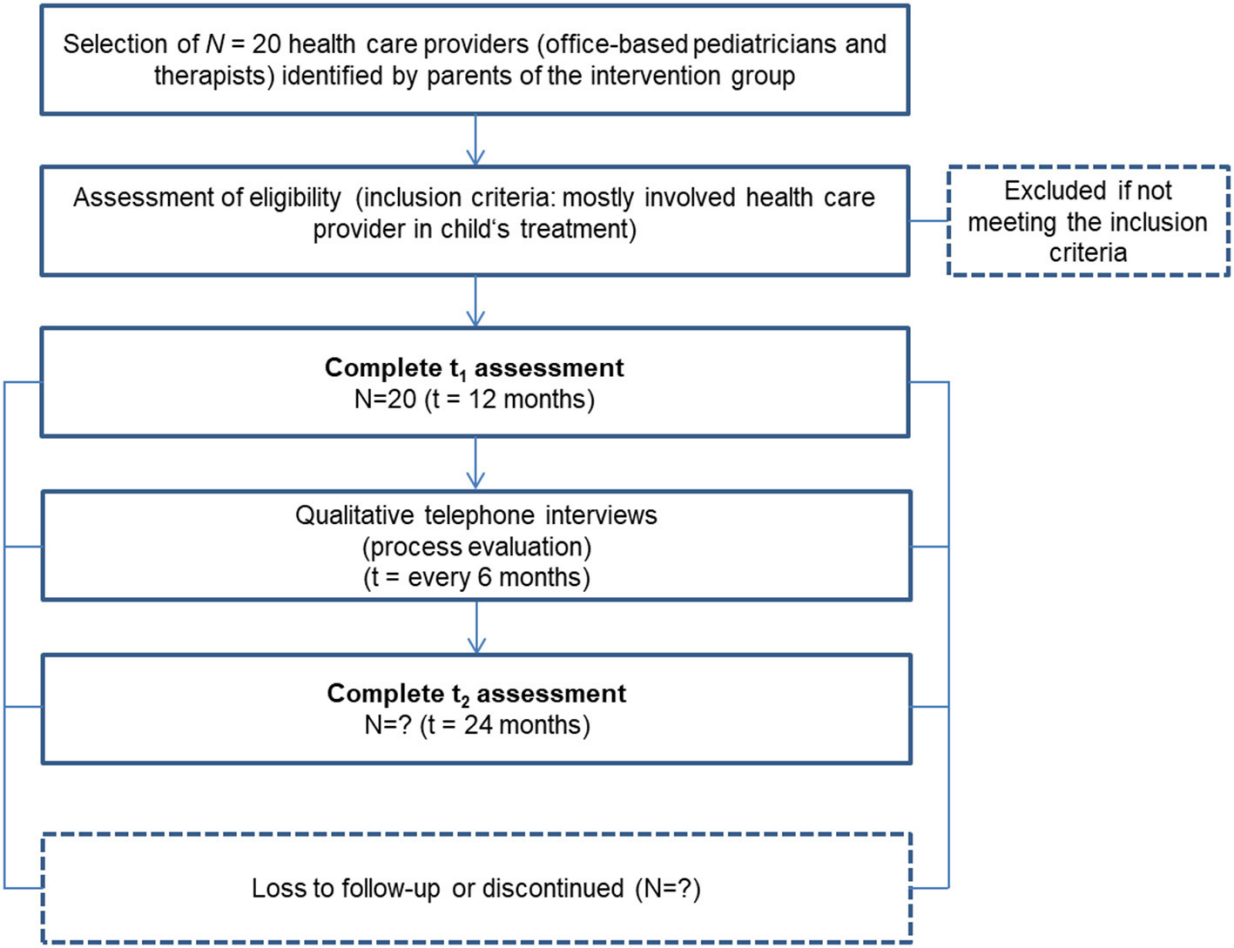
Participant timeline of healthcare providers in phase 3.

Additionally, we are conducting interviews with participants analyzing interview data on those caregivers' (*N* = 2 x 25) and *N* = 20 healthcare providers' acceptance of the CM intervention in the process-evaluating part of this phase. Inclusion criteria and sample sizes pursued are shown in Figures 2, 3.

A cost-effectiveness analysis determines the costs associated with the intervention.

### Intervention and Procedures

#### Phase 1—PICS Testing (41)

The *PICS* consists of 19 validated core items and 5 scales (*access to care, care goal creation/planning, family impact, communication between healthcare provider and family, team functioning/performance/quality/connectivity*). In the *PICS*, the questions are structured to refer to the overall experience of the family through the entire continuum of care and not just care in a single setting or at one time. For this reason, the authors propose a 12-month assessment period to evaluate care integration by care team members. There are supplementary questions that have not been validated due to sample size limitations (41) as well as accompanying demographic and healthcare status questions. Due to our focus on improving integrated care with a CM, we included the items of “Module 3: Integrator” in addition to the already validated core set of items. Our translation follows the six steps in the “Functional Assessment of Chronic Illness Therapy Translation Procedures and Guidelines” (FACIT) (42). Content validity is tested in cognitive pilot interviews with caregivers of children with different chronic diseases (*N* = 10). In the cognitive interviews, language, comprehensibility, unambiguity, and relevance/significance of the individual items are tested (43). The instrument is adapted after seven interviews based on previous results, and another three interviews are conducted. *N* = 400 caregivers of children with chronic diseases serve as the population for psychometrically evaluating the adapted instrument. Psychometric testing is conducted in anonymized form since there is no need of identifying individual persons.

#### Phase 2—Multi-Perspective State Analysis

**Figure 4:**
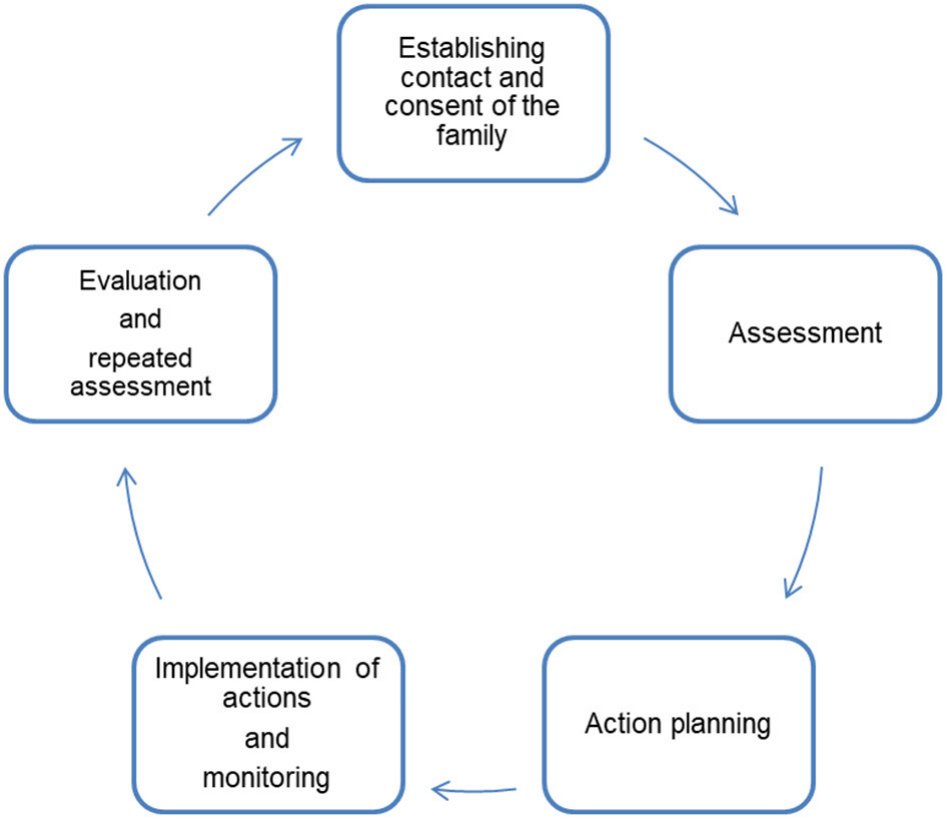
Five steps of Case Management [modified after Wendt (33)].

This phase includes a description of the activities carried out by caregivers in the context of integrated care as well as an evaluation of the quality of integrated care from the perspective of caregivers and healthcare providers involved in treating SMA I and II patients. The qualitative and quantitative methods listed below are used to describe and analyze the healthcare situation of patients medicated in the neuromuscular centers in Freiburg, Essen, and Tuebingen:

1. *Semi-Structured (Telephone) Interviews With Caregivers of Children With SMA I and II*
  Semi-structured (telephone) interviews are conducted with caregivers of children with SMA I and II, in which their experiences with their children's current care situation are investigated. The semi-structured interviews either take place at the SMA patient's regular inpatient stay or are conducted by telephone. We referred to the “life-course theory” (LCT) as a theoretical framework in developing the interview guide (44). The LCT is suitable because it focuses on predictable transitions in the lives of patients, the family context, and aspects of stress and resilience. Participants are selected applying a purposeful sampling strategy to capture the broadest possible spectrum of experiences (45). The selection of interview partners is guided by contrasting characteristics, such as the patient's age, health condition/stage of the disease, family situation, and geographical location. Depending on the interim results, further contrasting characteristics are possible (46). The interviews are digitally recorded following permission by the participants. This interview is scheduled to last 60 min and is conducted by a psychologist of the project team.
2. *Description and Analysis of Families' Activities Concerning Integrated Care*
  The activities of the same selected caregivers in the care-coordination context are documented themselves over a 4-week period with help from a self-developed care diary. The latter involves a tabular documentation form that can be used to document the type, duration, timing, and satisfaction with an activity's results. At the same time, the caregivers can indicate whether they could delegate the activity or whether they had to do it themselves. An additional file shows the care diary in more detail [see Supplementary Material]. In this way, it is possible to describe the allocation of coordination activities and the indirect costs incurred. The care diaries are pseudonymized and sent to the participants by mail. Participants are asked by telephone to return their care diary at regular intervals.
3. *Semi-Structured Telephone Interviews With Selected Healthcare Providers of Patients*
  Semi-structured telephone interviews are also conducted healthcare professionals significantly involved in the treatment of children with SMA I or II. The interviews focus on the experiences of the healthcare providers caring for patients with SMA, especially the perceived benefits and limitations in the cooperation with other healthcare providers. As we take a high degree of variability concerning the treatment period and region into account, we recruit office-based pediatricians, but also physiotherapists, occupational therapists, speech therapists, etc. The interviews are digitally recorded with the participants' permission. The interview is scheduled to last 15-30 minutes and is conducted by a psychologist of the project team.
4. *Conception of CM*
  Our CM concept builds on established service models, e.g., CM to support families after discharge from hospital of premature infants (32, 33). A certified pediatric nurse and certified CM assistant will take on the role as CM in the project. The CM will assess the care situation in its entirety together with the families in order to identify areas of sub-standard care and insufficient care integration. This goal will be achieved by the following activities (Figure 4):
  - Regular, structured conversation with participants regarding the overall care including the organization of specialist appointments, provision of medical aids, the child's integration in pre-school and school-activities, family support
  - Support in organizing and coordination of appointments within the medical center, i.e., combination of appointments with different specialists to avoid repeated visits to the hospital
  - Supporting information exchange between providers in and outside the hospital who do not communicate with each other on a regular basis, e.g., physiotherapists
  Within this established description of a CM service, we plan to tailor the service to the specific needs of patients with SMA, their caregivers and care providers based on the findings of the multi-perspective state analysis/phase 2. We hypothesize that the following aspects specific SMA could play a role for the conceptualization of the CM:
  - As SMA is a rare condition caregivers usually have little knowledge about the disease and a high demand for information regarding requiring input from a variety of care providers especially in the early stage after diagnosis.
  - SMA manifests early in a child's life and has a severe impact on patient and family. During this phase, caregivers are in need of continuity and support. Experienced caregivers however, may differ in their needs, e.g., the child's care situation could be medically stabile but the transition from one school to another might be challenging psychologically for the patient and family.
  - Since the approval of Nusinersen as a disease-modifying treatment in 2017, two other drugs have been approved for the treatment of SMA (Risdiplam and Onasemnogene abeparvovec). Caregivers are facing challenging treatment decisions for which they may benefit from counseling by several care providers, i.e., pediatric neurologist, physiotherapist, psychologist, etc.
  - Recent studies investigating the outcome under treatment with the above-mentioned drugs suggest a change of the SMA phenotype. As an example, patients frequently develop a severe scoliosis in early years. An effective collaboration among specialists and coordinated communication with caregivers seem particularly important.

Based on the findings of the multi-perspective state analysis the research team will develop suggestions for the needs-based tailoring of the CM-concept. Applying a participatory approach, we will discuss the concept with participating caregivers and care providers to elicit feedback and modify the concept as needed prior to phase 3.

#### Phase 3—Main Study

Qualitative and quantitative methods are applied to describe the effects of CM and the digital platform and to gather the chosen outcomes. The quantitative methods are implemented within the framework of a pre-post comparison at three points in time. The chosen primary outcomes for caregivers are measured before the intervention (baseline measurement, t0), 12 months after the intervention (t1) and, after a 24-month follow-up period (t2). In the control group, assessment time points are analogous, t0 being the time when the respondents were recruited to participate in the study. At t0, caregivers are sent paper-pencil pseudonymized questionnaires on chosen primary outcomes and sociodemographic information. After t0-data collection, the intervention is implemented in the intervention group. To test for 12- and 24-month sustainability (t1, t2) of the effects of the intervention the caregivers receive follow-up questionnaires on primary outcomes identical to the baseline measurement. Participants are asked by telephone to return the questionnaires at regular intervals.

1. *Semi-Structured Interviews With Caregivers on Process Evaluation and Secondary Outcomes*
  In addition to the questionnaires, semi-structured telephone interviews take place at five points in time (every 6 months) in both groups. Upon receipt of the filled t0-questionnaires, telephone appointments are arranged with the caregivers. In the telephone interviews, selected secondary outcomes related to caregivers and child are investigated. In the intervention group, telephone interviews also function to explore the CM process and digital-platform use. We ask the caregivers about their acceptance and perception of the intervention's feasibility (e.g., which factors are helpful, which are barriers) concerning their child's everyday care. The interviews are digitally recorded with the participants' permission of the participants. This interview is scheduled to last 15 min and is conducted by a psychologist of the project team.
2. *Process Evaluation of the CM by Involved Healthcare Providers*
  The CM and digital-platform experiences of selected healthcare providers are studied by questionnaire surveys and semi-structured interviews. The latter are conducted in the middle and end of the intervention phase (t1, t2) so that the respondents have as much experience with the CM as possible. Healthcare providers are asked to give us their assessment of the intervention (e.g., its usefulness, suggestions for improvement). This interview is scheduled to last 15 min and is conducted by a psychologist of the project team.

### Study Measurements and Outcomes (Main Study)

Outcome measurement primarily focuses on the affected families' perspective, which is why their perceptions and data are our primary endpoints. The healthcare providers' perspectives as well as changes in their child's health status or quality of life are secondary endpoints. Caregiver-related primary outcome measures are assessed via self-administered paper-pencil questionnaires incorporating already validated instruments.

#### Primary Outcomes

CM is primarily designed to support and relieve caregivers in caring for their child. To operationalize this purpose, we are examining two primary outcome parameters:

1. *The quality of integrated care from the caregivers' perspective*
  To assess the intervention's influence on the caregiver-evaluated quality of integrated care, we are administering the *PICS* questionnaire validated in phase 1 for German-speaking countries. Higher scale values indicate better-perceived care integration (41). We expect higher mean scale values after the intervention compared to baseline measurements. We also expect higher mean scale values in the intervention group than in the control group.
2. *The Health-Related Quality of Life of Caregivers*
  Two questionnaires are applied to assess caregivers' health-related quality of life:
  - *Familien-Belastungs-Fragebogen (FaBel-Fragebogen)* (47)
    The *FaBel* questionnaire is administered to determine how parents themselves assess the family burden caused by disease in children and adolescents. It is based on the *Impact on Family Scale* translated into German and psychometrically tested (48). It contains 33 Likert-scaled items to capture the daily social burden of caregivers, the personal burden, burden of siblings, the financial burden, problems coping with the burden, and a total score of the overall burden. Higher scale values indicate a heavier burden. We expect lower mean scale values after the intervention compared to the baseline measurement. We also expect lower mean scale values in the intervention group than in the control group.
  - *Short-Form-Health-Survey [SF-12, short version of SF-36* *(* *49* *)* *]*
    The SF-12 is administered to assess the caregiver-reported health-related quality of life during the past 4 weeks. It is a generic 12-item questionnaire that supplies a subjective health status summary score of physical and mental health. This total value results from four health components each: Physical health comprises *general health, physical functioning, role limitations due to physical health problems and bodily pain*; subjective mental health comprises *vitality (energy/fatigue), social functioning, role limitations due to emotional problems and mental health*. Higher scale values are indicative of better health. We expect higher mean scale values after the intervention compared to the baseline measurement. We also expect higher mean scale values in the intervention group than in the control group.

#### Secondary Outcomes

Giving relief to families should subsequently lead to improved quality of care and better health status of the child. Concerning the child with SMA, we gather information on medical and therapeutic care (planned and unplanned) as well as on the supply and use of aids via recurring telephone interviews with caregivers. In addition, the child's quality of life is assessed by their caregivers using the *PedsQL*™ *4.0 SF15* (50). The Likert-scaled 15-item questionnaire is a shortened version of the 23-item *PedsQL*™ *4.0 Generic Core Scales* that was developed to measure generic pediatric health-related quality of life concerning the past 4 weeks. We use the adult report standard versions for toddlers (2-4 years of age) and young children (5-7 years of age) as the participating SMA patients are within this age range. The questionnaire contains four scales that cover the parent-reported problems of the child in *physical functioning, emotional functioning, social functioning*, and *school functioning*. Caregiver-related, secondary outcomes are information on work absenteeism due to illness and the use of medical and therapeutic services. The healthcare provider-related secondary endpoint is the reported experience concerning the CM.

### Data Management and Data Analysis

Data entry is being mainly done by certified research assistants and project coordinators. Standard processes (i.e., verifying that data is within an expected range of values) are implemented to improve the accuracy of data entry. All personal project data is only accessible to selective project coordinators and will be deleted 36 months after the end of the project. Personal data are also kept separate from scientific data. Under the assumption of missing data in the questionnaires, multiple imputations are envisaged for respective analyses. The study population's demographics are reported descriptively.

#### Phase 1—PICS Testing

The statistical methods used in psychometric testing of the *PICS* rely on the procedure in the original publication and are supplemented by analyzing further properties (41). Distribution properties and internal consistency (Cronbach's alpha) are determined. The factor structure identified in the original questionnaire is examined *via* structural equation models (structural validity). Furthermore, we test hypotheses to investigate construct validity (51). In addition to the psychometric properties tested for the original version, the instrument's fit to the Rasch model's requirements is also assessed (52). The IBM SPSS® and IBM AMOS® statistical software packages are used for data analysis, as is WINSTEPS (for Rasch analyses).

#### Phase 2—Multi-Perspective State Analysis

Evaluation of the care diaries is statistically descriptive. Based on a content-analytical procedure, categories are formed and the average satisfaction with, as well as the average time spent on the individual coordination activities, are benchmarked.

For qualitative analyses of the interview data, audio files are transcribed by an external service provider and the transcripts are subsequently analyzed using qualitative content analysis. MAXQDA® is used as a data management program.

#### Phase 3—Main Study

The semi-structured interviews are processed in the same way as in phase 2.

Because we are investigating a rare disease, too few participants can be enrolled to enable the statistical significance of our hypothesis to be proven. Instead, our statistical analysis focuses on the descriptive determination of effect sizes (ES):

1. *Determining ES of the Change in the Intervention Group Between t0 and t1/t2*
  We assume that between t0 and t1, we detect an at least medium effect according to the usual conventions (ES ≥ 0.4) and that this effect tends to persist over the long term (after 24 months) (ES ≥ 0.3). In terms of sensitivity analyses, different variants of ES (standardized effect size, standardized response mean) are applied. Our hypotheses will be considered confirmed if the expected ES is obtained for all variants. We calculate confidence intervals for ES.
2. *Determining ES of the Pre-Post Difference in the Intervention and Control Group*
  A variance analysis with interaction effect (intervention vs. control group) is done and the partial eta squared (η^2^) is considered as ES. The hypothesis is tested whether η^2^ is at least medium according to the usual conventions (η^2^ ≥ 0.06).

These analyses are performed separately for primary and secondary outcome parameters. Based on our results, we conduct a power analysis to calculate the number of participants that would be required for a confirmatory evaluation study that might be performed in follow-up to detect the intervention's superiority over “usual care” in terms of statistical significance.

In addition, we analyze the intervention's cost-effectiveness by determining three economic outcome measures:

1. *Costs of the Intervention*
  The costs of the intervention consist primarily of personnel expenses incurred for CM. For this purpose, the time spent on activities for integrated care is documented and a distinction made between “direct contact with patients and healthcare providers” and “activities without direct contact.” The costs are determined by the ratio of time expenditure and standard wages per patient/year. Added to this are annual maintenance costs of the electronic platform per patient/year taken into account.
2. *Costs in the Context of Patient Care*
  The costs arising from medical and therapeutic services are collected based on the families' self-reporting and are determined by standardized unit costs. This involves investigating services for patients with SMA, such as visits to the doctor, medication, therapies, and aids. Furthermore, incurred costs associated with caregivers' absenteeism at work due to illness are determined.
3. *Costs of Informal Care Activities by Families (Care and Care Coordination)*
  The costs of informal family care are captured for selected periods. This involves daily care for the child as well as coordinating activities according to the amount of time spent. The contribution to costs is based on the employment relationship of the respective caregiver.

## Discussion

Much research progress has been made in investigating the genetic basis of SMA, its clinical features, and its care options. Less priority has been given to gaining insight into the impact on the lives of affected families and their care management (14). Studies show that the care situation for patients with chronic, complex conditions is heterogeneous: Some families seem to be receiving support already from a care coordinator or similarly functioning person within care management, others find themselves on their own (24, 53). The caregivers of patients with SMA I and II are confronted with highly challenging coordination and organization factors in their daily lives to ensure good care for their children. Continuous and patient-centered care coordination is essential to guarantee a consistent care plan among numerous healthcare providers and to ensure the healthcare system is organized efficiently (54). Across the ever-changing care situation of patients with SMA I and II, and to support their caregivers, we need to revise previous care models. New care models need to be tested in practice and systematically evaluated based on input from affected families and involved healthcare providers. The clinical embedding of our CM program and medical expertise of the Case Manager ensure that care is provided in accordance with guidelines. Regular assessments could enable a rapid specialist consultation to be initiated in the event of newly occurring problems such as swallowing difficulties. In this way, CM could enhance patient safety, as a deterioration in the child's state of health would be detected at an early stage. The continuous availability of CM, also beyond planned outpatient consultations at the neuromuscular center, could facilitate the prompt detection of psychosocial stress in the family as well as a reaction to it, and would contribute to empowering the caregivers. Another strength is the participatory research method through a demand-oriented approach to CM based on the practical experience of caregivers and healthcare providers.

At the end of the project, evidence-based data on quality and effectiveness as well as on the costs of the intervention developed will be available. An independent, open evaluation of this intervention combined with qualitative data will enable us to detect possible barriers and to improve CM based on feedback. By knowing the expected improvements as a result of the intervention, the effect size can be estimated for future cluster-randomized studies (which in the case of rare diseases require the participation of many centers); hence a sound power calculation can be conducted. In order to enable transferability, we will provide a detailed description of the CM's tasks and its relation to the measured outcomes in the presentation of the results at the end of the study.

This study has some limitations. Low patient numbers due to the rarity of disease allow only rough estimates of effect sizes; hence we cannot rule out any over- or underestimated effects. Even if we can assume that the care situation of SMA patients in Germany is largely regionally independent, recruitment is mainly limited to families whose children are medicated in the neuromuscular centers of Freiburg, Essen, and Tuebingen. Furthermore, a fluctuating diagnosis within the SMA spectrum renders our study's inclusion criteria (only patients with SMA I and severe II) too rigid. Experience shows that the clinical manifestations of different subtypes are similar, so caregivers of children with SMA III-IV are often confronted with similar problems. Further research with the involvement of many participating centers will be necessary to provide individual care solutions nationwide.

Our disease-specific orientation and multidimensional approach in development reveal the expectation that our intervention for this patient group could also be applied in other regions in Germany after minor adaptation. Once its effectiveness has been proven, the experience with this CM approach gained during this study may also benefit care strategies in other (non-pediatric) patient groups with rare diseases and/or chronic complex courses.

## Ethics Statement

The studies involving human participants were reviewed and approved by Ethics-Commission Albert-Ludwigs-University Freiburg. The patients/participants provided their written informed consent to participate in this study.

## Author Contributions

JW: drafting and revision of the manuscript. TL and EF-G: initiation, conception, design, and coordination of the research project. TL, JW, and EF-G: development of the intervention and evaluation materials. TL: implementation of the intervention. All authors read and approved the final version of the manuscript.

## Conflict of Interest

The authors declare that the research was conducted in the absence of any commercial or financial relationships that could be construed as a potential conflict of interest.

## Publisher's Note

All claims expressed in this article are solely those of the authors and do not necessarily represent those of their affiliated organizations, or those of the publisher, the editors and the reviewers. Any product that may be evaluated in this article, or claim that may be made by its manufacturer, is not guaranteed or endorsed by the publisher.

## Abbreviations

CM: case management
ES: effect size
FaBel: Familien-Belastungs-Fragebogen
LCT: life-course-theory
PICS: Pediatric Integrated Care Survey
SF-12: 12-item Short-Form-Health-Survey
SMA: spinal muscular atrophy
SMA-C+: name of the overall project, in which the study took place (“SMA” for spinal muscular atrophy; “C” for CM; “+” for adding of an IT component to CM)
SPC: social pediatric center.
