# Supplementary material for: Evaluation of a Case Management to Support Families With Children Diagnosed With Spinal Muscular Atrophy—Protocol of a Controlled Mixed-Methods Study
Source: Front Pediatr. 2021 Aug 3;9:614512. doi: 10.3389/fped.2021.614512 (PMC8369478; doi:10.3389/fped.2021.614512)
Supplement: Supplementary file 1 [file Data_Sheet_1.PDF]

CARE DIARY FOR CAREGIVERS

Dear Family________________________,

first of all, thank you very much for supporting us in our research project! The aim of the project is to improve the interaction between persons involved in treatment in order to increase the quality of care for patients with SMA 1 and 2 and to relieve the burden on affected families. To achieve this, it is important for us to find out about the extent to which you are involved in the care of your child. With this care diary, we ask you to document the **organizational activities** you have carried out to ensure that your child receives good health care. You will find examples of these activities on the back of this template.

Please complete the diary for a total of 4 weeks (28 days). First, please enter your documentation period in the top line of the form (p. 3) (i.e. start and end date; e.g. 06.04.2020 **-** 04.05.2020).

Then please enter for **each day**:

- **which activity** you have performed,
- **how long** it lasted (in minutes),
- whether you are **satisfied with the result** (according to school grades: 1 = very good to 6 = unsatisfactory)
- which would **help you to do this task better** and whether **another person could take over the task** to relieve you.

After completing the form, send the pages 3-10 stapled together without these instructions and without any further details about yourself (for reasons of anonymity) in the enclosed free return envelope to the Section of Health Care Research and Rehabilitation Research.

Many thanks for your effort and cooperation!

EXAMPLES:

Date	Activity (organization or coordination)	Duration (approxi-mately in minutes)	Satis-faction with the result (school grade)	What would help to improve it?	Could another person take over this task? If so, who?
7-5-2019	Postponed appointment pediatrician	14	2	Easier access to the practice	no
7-6-2019	Write an appeal against the refusal of the health insurance	30	3	Formulation support from other parents	Not really, but support through self-help would be good
7-8-2019	Drive to physiotherapy	60	1	Practice in the neighbourhood	no
7-10-2019	Internet research on new treatment options	45	3	Information on the reliability of sources	No, but a competent partner for discussion would be good

Please document **all** activities that come to your mind - even if you are not sure whether your activity is "organizational" or not.

If there are no activities on a particular day, you do not need to include this day in the list.

2	0	1		

CARE DIARY FOR THE PERIOD: ______________ - ______________

Date	Activity (organization or coordination)	Duration (approxi-mately in minutes)	Satis-faction with the result (school grade)	What would help to improve it?	Could another person take over this task? If so, who?

2	0	1		

Date	Activity (organization or coordination)	Duration (approxi-mately in minutes)	Satis-faction with the result (school grade)	What would help to improve it?	Could another person take over this task? If so, who?

2	0	1		

Date	Activity (organization or coordination)	Duration (approxi-mately in minutes)	Satis-faction with the result (school grade)	What would help to improve it?	Could another person take over this task? If so, who?

2	0	1		

Date	Activity (organization or coordination)	Duration (approxi-mately in minutes)	Satis-faction with the result (school grade)	What would help to improve it?	Could another person take over this task? If so, who?

2	0	1		

Date	Activity (organization or coordination)	Duration (approxi-mately in minutes)	Satis-faction with the result (school grade)	What would help to improve it?	Could another person take over this task? If so, who?

2	0	1		

Date	Activity (organization or coordination)	Duration (approxi-mately in minutes)	Satis-faction with the result (school grade)	What would help to improve it?	Could another person take over this task? If so, who?

2	0	1		

Date	Activity (organization or coordination)	Duration (approxi-mately in minutes)	Satis-faction with the result (school grade)	What would help to improve it?	Could another person take over this task? If so, who?

2	0	1		

Date	Activity (organization or coordination)	Duration (approxi-mately in minutes)	Satis-faction with the result (school grade)	What would help to improve it?	Could another person take over this task? If so, who?
